# Treatment of psychotic symptoms in bipolar disorder with aripiprazole monotherapy: a meta-analysis

**DOI:** 10.1186/1744-859X-8-27

**Published:** 2009-12-31

**Authors:** Konstantinos N Fountoulakis, Xenia Gonda, Eduard Vieta, Frank Schmidt

**Affiliations:** 1 Third Department of Psychiatry, Aristotle University of Thessaloniki, Thessaloniki, Greece; 2 Department of Pharmacology and Pharmacotherapy and Department of Psychiatry Kutvolgyi Klinikai Tömb, Semmelweis University, Budapest, Hungary; 3 Bipolar Disorders Program, Hospital Clinic, University of Barcelona, IDIBAPS, CIBERSAM, Barcelona, Spain; 4 Tippie College of Business, University of Iowa, Iowa City, IA, USA

## Abstract

**Background:**

We present a systematic review and meta-analysis of the available clinical trials concerning the usefulness of aripiprazole in the treatment of the psychotic symptoms in bipolar disorder.

**Methods:**

A systematic MEDLINE and repository search concerning clinical trials for aripiprazole in bipolar disorder was conducted.

**Results:**

The meta-analysis of four randomised controlled trials (RCTs) on acute mania suggests that the effect size of aripiprazole versus placebo was equal to 0.14 but a more reliable and accurate estimation is 0.18 for the total Positive and Negative Syndrome Scale (PANSS) score. The effect was higher for the PANSS-positive subscale (0.28), PANSS-hostility subscale (0.24) and PANSS-cognitive subscale (0.20), and lower for the PANSS-negative subscale (0.12). No data on the depressive phase of bipolar illness exist, while there are some data in favour of aripiprazole concerning the maintenance phase, where at week 26 all except the total PANSS score showed a significant superiority of aripiprazole over placebo (d = 0.28 for positive, d = 0.38 for the cognitive and d = 0.71 for the hostility subscales) and at week 100 the results were similar (d = 0.42, 0.63 and 0.48, respectively).

**Conclusion:**

The data analysed for the current study support the usefulness of aripiprazole against psychotic symptoms during the acute manic and maintenance phases of bipolar illness.

## Background

The treatment of bipolar disorder (BD) is difficult since the illness itself is complex [1-7]. In the BD clinical picture, psychotic features are a very frequent manifestation although they are not considered to constitute a core feature of the disorder. Delusions are relatively more common than hallucinations. However, it is reported that unipolar-depressed patients who later 'convert' to BD over time, as well as bipolar depressives, manifest more frequently psychotic features and pathological (psychotic) guilt [8,9]. Additionally, within the BD patient group it has been suggested (but not proven) that those patients with a history of psychotic symptoms suffer from a greater impairment regarding the neuropsychological performance especially concerning verbal memory and executive function [10,11].

Psychotic features include delusions and hallucinations and both can be mood congruent or non-congruent depending on their content. Mood congruent psychotic features include those entirely consistent with the thought content (either manic or depressive) while mood incongruent features are largely unrelated to thought content. Overexaggerated thoughts of guilt, sin, worthlessness, poverty and somatic health, or on the contrary thoughts of exceptional mental and physical fitness or special talents, wealth, some kind of grandiose identity or importance are mood congruent delusions, and even persecutory ideas or ideas of reference when in accord with the thought content can be considered to be mood congruent. Non-congruent delusions include nihilistic delusions (Cotard delusion or Cotard syndrome, negation delusion), bizarre delusions and sometimes the delusions can be so excessive that the identity itself changes. Psychotic symptoms have a profound effect on insight especially in depressive episodes which otherwise are characterised by a fair degree of insight. Psychotic features and the lack of insight might lead to the refusal of any treatment and to the need for an involuntary admission to a hospital.

Only during the last few years have antipsychotics and especially atypicals or second-generation antipsychotics (SGAs) gained a position in the treatment of BD [12,13]. Their efficacy against acute mania is reported to be independent of sedation or of their effect on psychotic symptoms. Olanzapine, risperidone, quetiapine, ziprasidone and aripiprazole are approved for the treatment of acute mania, quetiapine and the olanzapine-fluoxetine combination are approved for the treatment of acute bipolar depression, and olanzapine, quetiapine and aripiprazole are approved for maintenance phase treatment.

Aripiprazole (7-(4-[4-(2,3-dichlorophenyl)-1-piperazinyl]butyloxy)-3,4-dihydro-2(1H)-quinolinone (OPC-14597), is a derivative of the dopamine autoreceptor agonist 7-(3-[4-(2,3-dimethylphenyl)piperazinyl]propoxy)-2(1H)-quinolinone (OPC-4392) [14,15], was developed by Otsuka in Japan and was first approved by the US Food and Drug Administration (FDA) in 2002 for the treatment of schizophrenia.

Although psychotic symptoms are common in bipolar patients, not all randomised controlled trials (RCTs) include their assessment and up to now there has been no review or meta-analysis on the efficacy of agents approved for the treatment of BD on these specific symptoms. The aim of the current review and meta-analysis was to focus on outcome measures of randomised controlled trial testing the efficacy of aripiprazole against psychotic symptoms in bipolar disorder. To the best of our knowledge no such analysis exists in the literature to date, and the reviews available [16-25] either do not include all the trials that have been conducted so far or do not focus on psychotic symptoms.

## Methods

### Search criteria

The first step of the search included a keyword search of Medline and the internet via Google with the words 'aripiprazole' and 'bipolar'.

The second step included search of the BMS site http://www.bms.com/clinical_trials/ as well as several relevant online repositories including http://clinicaltrials.gov, http://www.clinicalstudyresults.org and http://www.cochrane.org. The third step included scanning of the reference lists of various review and meta-analysis papers [21-28].

### Types of studies

The studies selected were RCTs with placebo or a comparator.

### Data extraction

All data were extracted by the same author (KNF) from the full published paper or the clinical study report synopsis. In some cases some of the data were extracted or calculated from published meta-analysis or reviews.

### Meta-analysis method

The following indices were calculated. Effect size (Cohen d) was calculated as the mean change divided by the standard deviation of the scale. It represents the difference between two groups in the amount of change.

Pooled standard deviation (SDp) was calculated by the following function:

Where *S*_p _is the pooled standard deviation, *n*_i _is the sample size of the i^th ^sample, *s*_i _is the standard deviation of the i^th ^sample, and *k *is the number of samples being combined.

The Q test for testing the homogeneity of studies was computed by summing the squared deviations of each study's effect estimate from the overall effect estimate, weighting the contribution of each study by its inverse variance. This was calculated only for the Young Mania Rating Scale (YMRS) and for placebo-controlled RCTs concerning the differential d versus placebo and for all studies concerning the absolute effect of aripiprazole. Not rejecting the homogeneity hypothesis leads to a fixed effects model because it is assumed that the estimated effect sizes only differ by sampling error, while the rejection of the homogeneity assumption leads to a random effects model that includes both intrastudy and interstudy variability.

The I^2 ^index measures the extent of true heterogeneity, and was calculated by dividing the difference between the result of the Q test and its degrees of freedom (k-1) by the Q value itself and multiplying by 100. This index can be interpreted as the percentage of the total variability in a set of effect sizes due to true heterogeneity, that is, to interstudy variability.

The Hunter-Schmidt meta-analysis software was used for the correction of the effect sizes (d) for sampling error and measurement error [29] concerning only the effect size which calibrated the difference between two groups in the amount of change (aripiprazole vs placebo).

### Acute mania/mixed episodes

Six trials assessed the efficacy of aripiprazole against acute manic/mixed episodes. Theses were CN138-009 [30], CN138-074 or NCT00036101 [31], CN138-135 or NCT00095511 [27], CN138-162 or NCT00097266 [32], CN138-007 which was negative and not published [33], CN138008 [34] and CN138-077 or NCT00046384, which did not produce any results due to the small number of patients recruited. Two of them (CN138-007 and CN138-077/NCT00046384) included a fixed dosage while the others included a flexible dosage design. Rapid cycling patients were excluded from CN138-135/NCT00095511. CN138-077/NCT00046384 did not produce any results due to the small number of patients recruited (29 in the aripiprazole arm and 27 in the placebo arm). Its design included also a fixed dosage, and it was prematurely closed because it was expected to produce negative results similar to CN138-007. All used YMRS as the primary outcome measure. Data on Positive and Negative Syndrome Scale (PANSS) is not reported by CN138009 and CN138007, while data for PANSS total only and some but not all subscales are reported by CN138074 and CN138135.

**Figure 1:**
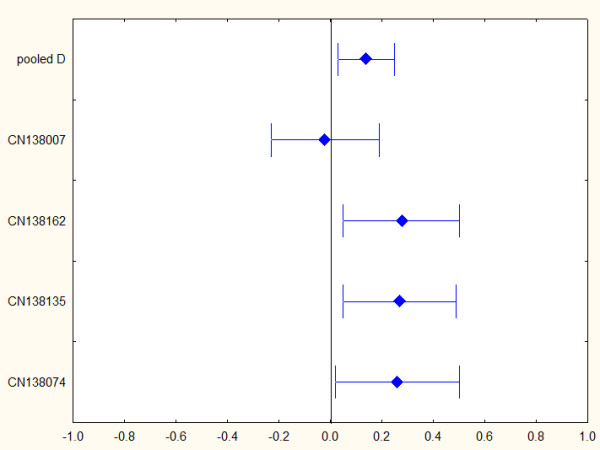
Forest plot of aripiprazole effect size against psychotic symptoms in acute mania (PANSS-total data).

The details of these studies (randomised patients, efficacy and safety sample, publications and results) are shown in Table 1. The baseline scores for all outcome scales are shown in Table 2. The similarity of baseline scores across trials and the similar pooled mean justified the pooling of all data concerning each arm across studies irrespective whether the specific study had a placebo or a comparator arm or not. The dropout rates are shown in Table 3. The changes in the PANSS scales scores are shown in Table 4. The effect sizes (d) are shown in Table 5. The side effects frequency is shown in Table 6. The forest plot is shown in Figure 1.

**Table 1:** List of acute mania trials of aripiprazole and their characteristics

Trial	Publication	Duration	COMP	PLC	Randomised, N			Efficacy sample, N			Safety sample, N			Results
						
					AR	COMP	PLC	AR	COMP	PLC	AR	COMP	PLC	
CN138-009	Keck *et al*., 2003 [27]	3 weeks	No	Yes									127	Agent > PLC
CN138-074/NCT00036101	Sachs *et al*., 2006 [28]	3 weeks	No	Yes	137		135	136		132	136		133	Agent > PLC
CN138-135/NCT00095511	Keck *et al*., 2009 [29]	12 weeks	Li	Yes	155	160	165	154	155	163	154	159	164	Agent = comparator > PLC
CN138-162/NCT00097266	Young *et al*., 2009 [30]	12 weeks	Hal	Yes	167	165	153	152	161	152	166	165	153	Agent = comparator > PLC
CN138-007	Unpublished	3 weeks	No	Yes	267		134	267 -		134 -	267 -		134 -	Agent = PLC
CN138-077/NCT00046384	Unpublished	3 weeks	No	Yes	29		27							No results
CN138008	Vieta *et al*., 2005 [32]	12 weeks	Hal	No	175	172		171	158		175	169		Agent > comparator
All RCTs														
Total by groups					1,060	497	746	1,005	474	704	1,025	493	711	
Total							2,303			2,183			2,229	
Only RCTs with PANSS data														
Total by groups					663	497	480	613	474	447	631	493	450	

Total							1,640			1,534			1,574	

Dash indicates missing data. AR = aripiprazole; COMP = comparator; PANSS = Positive and Negative Syndrome Scale; PLC = placebo; RCT = randomised controlled trial.

**Table 2:** The baseline scale scores in aripiprazole randomised controlled trials (RCTs) of acute mania.

Trial	PANSS-total	PANSS-positive	PANSS-negative	PANSS-cognitive	PANSS-hostility	N (efficacy sample)
Aripiprazole						
CN138-009	-	-	-	-	-	125
CN138-074	61.8 ± 16.7	-	-	-	-	136
CN138-135	62.0 ± 13.7	-	-	15.2 ± 3.4	10.1 ± 3.4	154
CN138-162	54.8 ± 10.3	16.0 ± 3.9	9.6 ± 2.6	14.7 ± 3.9	9.7 ± 2.6	152
CN138-007	-	-	-	-	-	267 -
CN138008	-	-	-	-	-	171
Pooled mean	59.5	16.0	9.6	14.9	9.9	1,005
Pooled SD	13.7	3.9	2.6	3.6	3.0	
Placebo						
CN138-009	-	-	-	-	-	123
CN138-074	62.5 ± 16.5	-	-	-	-	132
CN138-135	63.9 ± 13.1	-	-	15.3 ± 3.6	10.4 ± 3.6	163
CN138-162	54.4 ± 10.3	16.4 ± 4.9	9.4 ± 2.5	14.9 ± 3.7	9.7 ± 2.5	152
CN138-007	-	-	-	-	-	134 -
CN138008						
Pooled mean	60.3	16.4	9.4	15.1	10.0	704
Pooled SD	13.4	4.9	2.5	3.6	3.1	
Comparator						
CN138-009						
CN138-074						
CN138-135	63.2 ± 12.9	-	-	15.6 ± 3.5	10.5 ± 3.5	155
CN138-162	54.1 ± 10.3	16.1 ± 3.9	9.5 ± 2.5	14.6 ± 3.9	9.4 ± 2.5	161
CN138-007						
CN138008	-	-	-	-	-	158
Pooled mean	58.6	16.1	9.5	15.1	9.9	474
Pooled SD	11.6	3.9	2.5	3.7	3.0	

Dash indicates missing data.

^a^Calculated from Suppes *et al*. 2008 [21]; data were not used for the calculation of the pooled mean because there is no data on the change.

PANSS = Positive and Negative Syndrome Scale.

**Table 3:** Dropout in aripiprazole randomised controlled trials (RCTs) for acute mania; data concern numbers of patients while the last row concerns rates

Trial	Aripiprazole								Placebo				Comparator							
	
	3 weeks				12 weeks				3 weeks				3 weeks				12 weeks			
	
	Total	LE	AE	CW	Total	LE	AE	CW	Total	LE	AE	CW	Total	LE	AE	CW	Total	LE	AE	CW
CN138-009	76	23	11	25					104	53	13	38								
CN138-074	58	12	12	34					64	28	10	26								
CN138-135	82	9	23	15	113	12	31	70	87	36	13	10	82	26	21	5	106	26	28	52
CN138-162	41	9	14	18	72	13	24	35	44	14	16	14	44	10	8	26	70	11	18	41
CN138-007																				
CN138008	41	-	17	-	86	30	32	24					77	-	53	-	122	10	84	28
All RCTs																				
Dropout (%)	40.38	9.35	10.43	16.23	56.81	11.53	18.24	27.04	52.46	22.98	9.12	15.44	42.83	11.39	17.30	9.81	62.87	9.92	27.43	25.53
Only RCTs with PANSS data																				
Dropout (%)	36.22	6.79	10.77	15.16	56.81	11.53	18.24	27.04	43.62	17.45	8.72	11.19	42.83	11.39	17.30	9.81	62.87	9.92	27.43	25.53

Dash indicates missing data.

AE = adverse events; CW = consent withdrawal or other; LE = lack of efficacy; PANSS = Positive and Negative Syndrome Scale.

**Table 4:** Change in Positive and Negative Syndrome Scale (PANSS) scores in acute mania aripiprazole randomised controlled trials (RCTs)

Trial	3 weeks					12 weeks					N (efficacy sample)
		
	PANSS-total	PANSS-positive	PANSS-negative	PANSS-cognitive	PANSS-hostility	PANSS-total	PANSS-positive	PANSS-negative	PANSS-cognitive	PANSS-hostility	
Aripiprazole											
CN138-009	-	-	-	-	-						125
CN138-074	-10.0 ± 18.2	-	-	-	-						136
CN138-135	-9.5 ± 17.4	-	-	-2.0 ± 4.6	-2.1 ± 4.6	-10.6 ± 19.4	-	-	-2.9 ± 5.7	-2.5 ± 4.6	154
CN138-162	-8.2 ± 12.9	-3.8 ± 5.1	-0.4 ± 2.6	-2.4 ± 3.9	-2.3 ± 3.9	-9.8 ± 14.2	-4.9 ± 5.2	-0.2 ± 2.6	-3.2 ± 5.2	-3.0 ± 3.9	152
CN138-007	-2 ± -^a^	-	-	-	-						267 -
CN138-008	-	-	-	-	-	-	-	-	-	-	171
Pooled mean	-6.5	-3.8	-0.4	-2.2	-2.2	-10.2	-4.9	-0.2	-3.0	-2.7	*1,005*
Pooled SD	16.3	5.1	2.6	4.3	4.3	16.9	5.2	2.6	5.5	4.3	
Placebo											
CN138-009	-	-	-	-	-						123
CN138-074	-5.3 ± 17.9	-	-	-	-						132
CN138-135	-4.9 ± 16.7	-	-	-0.9 ± 4.8	-1.0 ± 3.6						163
CN138-162	-4.7 ± 12.4	-2.4 ± 4.9	-0.1 ± 2.5	-1.5 ± 6.2	-1.2 ± 6.2						152
CN138-007	-2.3 ± -^a^	-	-	-	-						134 -
CN138-008											
Pooled mean	-4.3	-2.4	-0.1	-1.2	-1.1						*704*
Pooled SD	15.8	4.9	2.5	5.5	5.0						
Comparator											
CN138-009											
CN138-074											
CN138-135	-7 ± 17.7	-	-	-1.6 ± 4.7	-1.6 ± 3.5	-7.4 ± 18.8	-	-	-2.0 ± 4.7	-1.8 ± 4.7	155
CN138-162	-8.8 ± 12.8	-4.2 ± 5.1	-0.3 ± 2.6	-2.5 ± 3.9	-2.6 ± 3.9	-11.7 ± 14.1	-5.4 ± 5.1	-0.3 ± 2.6	-3.9 ± 5.1	-3.5 ± 3.9	161
CN138-007											
CN138-008	-	-	-	-	-	-	-	-	-	-	158
Pooled mean	-7.9	-4.2	-0.3	-2.0	-2.1	-9.6	-5.4	-0.3	-3	-2.7	*474*
Pooled SD	15.4	5.1	2.6	4.3	3.7	16.6	5.1	2.6	4.9	4.3	

Dash indicates missing data.

^a^Not included in the calculation of the pooled measures because of a different study design (fixed dosage).

**Table 5:** Effect sizes for Positive and Negative Syndrome Scale (PANSS) total and subscales

	PANSS total		PANSS-positive		PANSS-negative		PANSS-cognitive		PANSS-hostility	
	
	d	95% CI	d	95% CI	d	95% CI	d	95% CI	d	95% CI
Aripiprazole vs placebo										
CN138-009	-		-		-		-		-	
CN138-074	0.26	0.02 to 0.50	-		-		-		-	
CN138-135	0.27	0.05 to 0.49	-		-		0.23	0.01- to 0.45	0.27	0.05 to 0.49
CN138-162	0.28	0.05 to 0.50	0.28	0.05 to 0.51	0.12	-0.11 to 0.34	0.17	-0.05 to 0.40	0.21	-0.01 to 0.44
CN138-007	-0.02	-0.23 to 0.19	-		-		-		-	
CN138-008	-		-		-		-		-	
Pooled mean	0.14	0.03 to 0.25	0.28	0.05 to 0.51	0.12	-0.11 to 0.34	0.20	0.04 to 0.36	0.24	0.08 to 0.39
Hunter-Schmidt d	0.18	0.176 to 0.184	-		-		0.20	-	0.24	-
Comparator vs placebo										
CN138-135	0.12	-0.10 to 0.34	-		-		0.15	-0.07 to 0.37	0.17	-0.05 to 0.39
CN138-162	0.33	0.10 to 0.55	0.36	0.14 to 0.58	0.08	-0.14 to 0.30	0.19	-0.03 to 0.42	0.27	0.05 to 0.49
Pooled mean	0.23	0.10 to 0.36	0.36	0.14 to 0.58	0.08	-0.14 to 0.30	0.16	0.01 to 0.32	0.23	0.07 to 0.38

Dash indicates missing data.

**Table 6:** Side effects profile of aripiprazole in comparison to placebo (difference percentage).

Trial	Target	Overall side effects	Anxiety	Agitation	Acathisia	Constipation	Headache	Hyperprolactinaemia	Insomnia	Nausea	Sedation
CN138-009	Mania		8	1	9	7	5	-6	6	13	15
CN138-074	Mania	6	2	0.4	13.1	6.5	0.2	-7		5.5	9.4
CN138-135	Mania				8	4.3	0.8			9.3	6.8
CN138-162	Mania	0.1	11.4		6.8		1.8		2.1		5.4
CN138008	Mania				11.4		10.9		13.7		
CN138-096	Depression	11	2.9	4.7	22.7	-1.5	-1.1		11.5	9.8	1.3
CN138-146	Depression	6	6.5	9.3	18.6	1.6	-2.3		7.7	6.6	1.1
CN138-010	Maintenance	6	-14.5	-10.8	6.6		7.8		-19.3	4.3	-7.2
CN138-134	Mania	8.3			13.2					5.2	

Negative results suggest the effect in question was more frequent in the placebo group.

After including only the RCTs reporting PANSS data (CN138009 and CN138007 were excluded), in total 1,640 patients were randomised and the total efficacy sample included 1,534 patients (613 in the aripiprazole group, 447 the placebo group and 474 in active control groups) while the safety sample included 1,574 (631 in the aripiprazole group, 450 in the placebo group and 493 in active control groups) (Table 1). The pooled dropout rate for the RCTs which reported PANSS data was 36.22% for aripiprazole, 43.62% for placebo and 42.83% for comparator at week 3, climbing to 56.81% and 62.87% at week 12 for aripiprazole and comparator, respectively. The dropout rates are shown in detail in Table 3.

The pooled d value for total PANSS score for all placebo-controlled studies with a fixed model was equal to 0.191 (95% CI 0.08 to 0.304), with a Q value equal to 5.024 (degrees of freedom (df) = 3; *P *= 0.170), which does not reject the homogeneity hypothesis. The I^2 ^is equal to 40.283, which means that less than half of observed variability in the effect sizes across studies is due to true heterogeneity. However a random model gives similar results (d = 0.194; 95% CI 0.05 to 0.34). The Hunter-Schmidt method reports d = 0.18 (95% CI 0.176 to 0.184) after correcting for sampling and measurement error (Table 5).

### Acute bipolar depression

There were two 8-week placebo controlled RCTs (CN138-096/NCT00080314 and CN138-146/NCT00094432) concerning the use of aripiprazole in acute bipolar depression, and were both negative at study endpoint [35]. These included non-psychotic bipolar depressives and thus do not report data on psychotic symptoms.

### Maintenance treatment

There was one placebo-controlled RCT (CN138-010/NCT00036348) that studied aripiprazole in the maintenance phase [36]. Patients were stabilised with 15 to 30 mg of aripiprazole for 6 to 18 weeks and then randomised to a 1:1 ratio to aripiprazole or placebo for an additional 26 weeks. The baseline mean YMRS score was 2.5 ± 2.8 for the aripiprazole group and 2.1 ± 2.3 for the placebo group. The Montgomery-Åsberg Depression Rating Scale (MADRS) baseline scores were 3.9 ± 3.5 and 4.5 ± 4.2, respectively. Only anticholinergics and lorazepam were allowed as concomitant medication. During the 26 weeks 71.1% of patients under placebo and 71.4% of patients under aripiprazole received at least one concomitant medication. The primary efficacy outcome was time to relapse for a mood episode. From a total of 633 patients initially screened and 206 of them who completed the stabilisation phase, 161 were randomised (83 to placebo and 78 to aripiprazole). A total of 39 patients (50%) under aripiprazole and 28 (34%) under placebo completed the 26 weeks of the trial. The mean aripiprazole daily dosage at the end of 26 weeks was 24.3 mg. The time to relapse was significantly longer for aripiprazole (*P *= 0.02) and the hazard ratio (HR) was 0.52 (95% CI 0.30 to 0.91).

For the PANSS total score, a numerical trend favoured aripiprazole over placebo at any time point. At week 26, the changes (mean ± SD) in PANSS total scores were 5.2 ± 14.57 for aripiprazole and 9.1 ± 13.24 for placebo (*P *= 0.077), for the PANSS cognitive subscale score were for aripiprazole, 0.8 ± 4.55; placebo, 2.5 ± 4.41; *P *= 0.014) and for the PANSS hostility subscale score for aripiprazole, 0.8 ± 2.73; placebo, 1.8 ± 2.65; *P *= 0.032). All except the total PANSS score showed a significant superiority of aripiprazole over placebo, with d = 0.28, 0.38 and 0.71, respectively.

The adverse events reported by aripiprazole-treated patients at an incidence ≥ 5% and twice the incidence of placebo during the maintenance phase were tremor (9.1%), acathisia (6.5%), vaginitis (6.4%), and pain extremity (5.2%). One aripiprazole-treated patient and one placebo-treated patient attempted suicide in the stabilisation and maintenance phases, respectively. There were no significant differences concerning the QTc, while aripiprazole-treated patients showed a significant drop in prolactin levels. Concerning weight gain, 13% of aripiprazole-treated patients put > 7% of weight in comparison to none in the placebo group.

This same trial (CN138-010/NCT00036348) was expanded and included also a 74-week placebo controlled extension phase [37], which included 66 of the 67 patients who completed the 26-week period. Unfortunately only 12 of them (5 in the placebo group and 7 in the aripiprazole group) completed the 74-week treatment period. The reasons for this high discontinuation rate varied and included lack of efficacy, side effects (very low percentage) and most importantly the very design and structure of the study (the study was closed by the sponsor when the prespecified number of relapses had been attained). Because of this and because detailed descriptive data are not reported, arriving at conclusions is very difficult. The mean dosage of aripiprazole at the end of the 74-week period was 23.6 mg daily. It is reported that 29 out of the 66 patients relapsed (16 out of the 39 in the aripiprazole group (41%), and 13 out of the 27 in the placebo group (48.1%)). The only difference concerned manic relapses (nine in the placebo group and six in the aripiprazole group). Again the YMRS score significantly differed between groups. The adverse events had a similar rate to the 26-week period.

In both the above reports the median survival time for the aripiprazole group was not evaluable, while the median survival time for placebo was 118 to 203 days depending on the clinical subpopulation. At week 100, the changes (mean ± SD) in PANSS total scores were 7.9 ± 10.61 for aripiprazole and 11.8 ± 8.31 for placebo (*P *= 0.10), for the PANSS cognitive subscale score were for aripiprazole, 1.5 ± 3.12; placebo, 3.3 ± 2.59 (*P *= 0.01) and for the PANSS hostility subscale score for aripiprazole, 1.2 ± 2.49; placebo, 2.3 ± 2.07 (*P *= 0.03). Again all except the total PANSS score showed a significant superiority of aripiprazole over placebo, with d = 0.42, 0.63 and 0.48, respectively [38].

The expansion of the 135-008 trial [34] to a further 14 weeks failed to provide any results because of a high dropout rate, while the extension of the CN138-135/NCT00095511 trial [27] for an additional 40 weeks (52 weeks in total) comparing aripiprazole to lithium without a placebo arm suggested aripiprazole equal to lithium in the maintenance against manic episodes. No PANSS data are reported concerning this extension phase.

## Discussion

The meta-analysis of the four trials that investigated the efficacy of aripiprazole on psychotic symptoms (assessed by the PANSS) during acute manic/mixed episodes suggests that the effect size versus placebo was equal to 0.14, but a more reliable and accurate estimation is 0.18 for the total PANSS score. The effect was higher for the PANSS positive subscale (0.28), PANSS hostility subscale (0.24) and PANSS cognitive subscale (0.20), and lower for the PANSS negative (0.12). The majority of these trials included patients with moderate to severe manic episodes, some of whom also had psychotic symptoms. No data on the depressive phase of bipolar illness exist, while there are some data in favour of aripiprazole concerning the maintenance phase, where at week 26 all except the total PANSS score showed a significant superiority of aripiprazole over placebo (d = 0.28 for positive, d = 0.38 for the cognitive and d = 0.71 for the hostility subscales) and at week 100 the results were similar (d = 0.42, 0.63 and 0.48, respectively). It is important to note that the efficacy for both haloperidol and lithium is similar to aripiprazole in the psychotic symptoms of BD. This could seem odd concerning lithium, which is not considered to possess antipsychotic efficacy (although it is recommended as augmentation strategy in refractory patients with schizophrenia). However the literature suggests that in essence lithium might exert a state-dependent effect on second messenger systems that is antidopaminergic-like during manic episodes (when dopaminergic activity seems to be elevated). Thus this state-dependent antidopaminergic activity could be responsible for this antipsychotic action [39,40].

In comparison to the baseline scores reported in schizophrenia RCTs with aripiprazole, the respective PANSS scores in bipolar RCTs are significantly lower and this is of course expected. The PANSS-positive scores in schizophrenia RCTs range are around 29, for PANSS-negative they are around 22 and for PANSS-hostility around 9.5, while difference from placebo is again larger with 2.63 points difference in PANSS-positive, 2.31 points for PANSS-negative and 1.96 for PANSS-hostility [41]. However in these studies no standard deviations are reported and it is not possible to derive, thus the real effect sizes can not be calculated [42]. The only comparison that can be made is in terms of the ratio change to baseline. This ratio is similar for PANSS-positive (around 10%), but much different concerning PANSS-negative (10% in schizophrenia vs 3% in BD) and PANSS-hostility (20% for schizophrenia vs 11% for BD).

These results should be interpreted in light of recent studies on common genetic findings in schizophrenia and mood disorders [43-47].

There are several meta-analyses in the literature concerning the efficacy of various agents in the treatment of bipolar illness, but none analyse the effect on psychotic symptoms [21-28]. These meta-analyses suggest that aripiprazole's antimanic effect is specific and not limited to control of agitation through sedation, but no data on psychotic symptoms are analysed. Additionally, there is a concern regarding aripiprazole and olanzapine maintenance data because the relevant studies included patients who were responders specifically to the drug under investigation during the acute phase.

A few issues concerning the data of the RCTs should be pointed out however, because they reveal the restrictions of RCTs, as well as the gaps in our current knowledge and understanding and treating of bipolar illness. The first point is that although acute mania is generally considered one of the 'easier to treat' psychiatric conditions, with only 5% of bipolar patients experiencing chronic mania (although such a diagnostic condition is not recognised by the Diagnostic and Statistical Manual, Fourth Edition, Text Revision (DSM-IV-TR)) [48], in the acute mania RCTs around half of acutely manic patients were non-responders at week 12, suggesting that they were not only chronic but maybe also refractory. The question whether the dosage should be raised is open and pressing. Higher dosages for all agents might be necessary to be studied. During the maintenance phase, around three-quarters of patients will drop out of aripiprazole treatment within the first year in comparison to almost all the patients under placebo, and although the difference is significant, it suggests that even under effective treatment, a significant number patients tend to relapse although at a lower frequency of episodes. Finally, the fixed dosage RCT was negative, suggesting that aripiprazole should be prescribed at an individualised basis. Similar issues have been recently raised because of the still unpublished results of the one study of paliperidone in acute mania, which also used a fixed dose approach (the second one utilised a flexible dosage design) and reported that 6 and 9 mg were not effective versus placebo while 12 mg was.

Conclusively, the data analysed for the current study supports the usefulness of aripiprazole against the psychotic symptoms during the acute manic/mixed and maintenance phases of bipolar illness, however there are specific issues the clinician should have in mind, such as that the maintenance effect is proven only in patients with an index manic episode that responded to aripiprazole during the acute phase. Higher and ever-increasing placebo rates constitute a problem of quality for RCTs today and limit the generalisability of results [49]. A limitation of this review is that most of the trials were sponsored by the pharmaceutical industry and were conducted to gain regulatory approval for aripiprazole for the treatment of bipolar disorder. Therefore, the possibility of sponsor bias induced in favour of their product cannot be excluded, especially since failed trials were not published and the available data from them are limited.

## Competing interests

KNF is member of the International Consultation Board of Wyeth for desvenlafaxine and has received grants or honoraria for lectures from AstraZeneca, Servier, Janssen-Cilag, Eli Lilly and research grants from AstraZeneca, Janssen-Cilag, Elpen and Pfizer Foundation. EV has acted as consultant, received grants, or received honoraria for lectures by the following companies: Almirall, AstraZeneca, Bial, Bristol Myers Squibb, Eli Lilly, Forrest Research Institute, GlaxoSmithKline, Janssen-Cilag, Jazz Lundbeck, Merck Sharpe Dohme, Novartis, Organon, Pfizer, Sanofi, Servier, UBC. FS has no conflicts of interest. XG has received support for travelling and lecturing by GlaxoSmithKline, Sanofi, Eli Lilly Organon, Servier and Richter
